# Exercise-Based Rehabilitation in Severe COVID-19 Survivors with Long COVID: A Randomized Controlled Pilot Study

**DOI:** 10.3390/medsci14020222

**Published:** 2026-04-29

**Authors:** Edson Fonseca Pinto, Nailton José Brandão Albuquerque Filho, Jéssica Costa Leite, Tatianne Moura Estrela Gusmão, Larissa Nayara de Souza, Roque Ribeiro da Silva Júnior, Maria Irany Knackfuss, Grasiela Piuvezam

**Affiliations:** 1 Post-Graduation Program in Public Health, Federal University of Rio Grande do Norte, Natal 59078-970, Brazil; gpiuvezam@yahoo.com.br; 2 Department of Physical Education, State University of Rio Grande do Norte—UERN, Mossoró 59625-900, Brazil; mariaknackfuss@uern.br; 3 Systematic Review and Metanalysis Laboratory (Lab-Sys/CNPq), Federal University of Rio Grande do Norte, Natal 59078-970, Brazil; 4 Department of Physical Education, Federal Rural University of Pernambuco—UFRPE, Serra Talhada 56909-535, Brazil; nailtonalbuquerquefilho@gmail.com; 5 Department of Physiotherapy, UNIFACISA University Center—Paraíba, Mossoró 59625-900, Brazil; jessica.leite@maisunifacisa.com.br (J.C.L.); tatianne.gusmao@maisunifacisa.com.br (T.M.E.G.); 6 Graduate Program in Health and Society—PPGSS, State University of Rio Grande do Norte, Mossoró 59625-900, Brazil; larissanay15@gmail.com; 7 Multicenter Graduate Program in Physiological Sciences (PPGMCF), Faculty of Health Sciences, State University of Rio Grande do Norte (UERN), Mossoró 59625-900, Brazil; roquejunior@alu.uern.br; 8 Department of Public Health, Federal University of Rio Grande do Norte, Natal 59078-970, Brazil

**Keywords:** exercise, quality of life, physical and rehabilitation medicine, post-COVID-19 syndrome, telerehabilitation

## Abstract

Introduction: Post-hospital rehabilitation is essential for survivors of severe COVID-19, as prolonged immobility and clinical severity often lead to muscle weakness, reduced cardiovascular capacity, and impaired respiratory function. Physical exercise during and after hospitalization may mitigate these effects and support functional recovery. This study aimed to evaluate the effectiveness of a physical exercise-based rehabilitation program in survivors of severe COVID-19. Methodology: A randomized clinical trial was conducted with 30 survivors allocated to two groups: multicomponent exercise (GEm) and multicomponent exercise combined with inspiratory muscle training (GEmTMI). The interventions were performed three times per week for 40–60 min. Quality of life, physical activity level, functional status, and physical capacity were assessed before and after six weeks. Results: Comparisons between GEm and GEmTMI showed significant differences in the 6 min walk test (6MWT) at baseline (*p* = 0.043) and in the Physical Activity Index (IPAQ) after the intervention (*p* = 0.002). When the total sample was analyzed, significant improvements were observed across all outcomes after rehabilitation, including quality of life (SF-36), functional capacity (PCFS), physical activity level (IPAQ), respiratory muscle strength, and additional functional tests. Notable improvements included SF-36 Physical Functioning (*p* = 0.006) and Social Functioning (*p* = 0.009), PCFS (*p* = 0.011), IPAQ (*p* = 0.012), and performance in the 6MWT, STS, STS-1min, TUG, handgrip strength, PEmax, and PImax (all *p* < 0.001). Discussion: Multicomponent physical rehabilitation, with or without inspiratory muscle training, produced significant gains in physical activity level, functional capacity, dynamic balance, neuromuscular fitness, respiratory muscle strength, and quality of life. These findings underscore the importance of structured post-ICU rehabilitation to support comprehensive physical and psychosocial recovery in survivors of severe COVID-19.

## 1. Introduction

In the past two decades, several viral diseases have emerged with varying degrees of virulence and transmissibility, posing substantial challenges to public health and healthcare systems worldwide [1], as reported by the World Health Organization (WHO). Among these diseases, coronavirus disease 2019 (COVID-19), caused by SARS-CoV-2, had a particularly devastating impact. As of 7 April 2024, the WHO reported 775,293,630 confirmed cases and 7,044,637 deaths globally. In Brazil, 37.5 million cases and 702.1 thousand deaths were reported [2].

Although the case-fatality rate of SARS-CoV-2 (2.6%) is lower than that of SARS-CoV (11%) and Middle East respiratory syndrome (MERS; 35–50%), COVID-19 has proven far more disruptive because of its high transmission rate, resulting in millions of deaths and overwhelming healthcare systems worldwide [3]. COVID-19 is an infectious disease that can cause severe acute respiratory syndrome and may progress to multi-organ failure [4]. Patients with severe disease frequently develop acute respiratory, neurological, and musculoskeletal manifestations, which can lead to substantial impairments in standing, mobility, and functional independence [5,6].

The literature indicates that many patients discharged after hospitalization for COVID-19 experience persistent symptoms, including fatigue, shortness of breath, cognitive impairment, and sleep dysfunction [7,8,9,10,11]. These symptoms have been reported in patients discharged with or without ICU admission, as well as in “long-haul” patients [10], including those who initially presented with mild to moderate COVID-19 [10]. Moreover, a substantial proportion of individuals develop what has been described as post-viral fatigue syndrome or “long COVID” [5]. Rehabilitation guidelines for post-COVID-19 patients have been released by the WHO and by several countries [12].

Persistent symptoms include fatigue, shortness of breath, joint pain, chest pain, headaches, myalgia, brain fog, and gastrointestinal complaints [7,13,14,15]. These symptoms may be exacerbated by the considerable stress associated with hospitalization, including infrequent and brief interactions with healthcare professionals, absence of family at the bedside, and limited information regarding disease course, treatment options, and prognosis [16]. Accumulating evidence suggests that COVID-19 survivors may develop multiple sequelae beyond pulmonary damage [17], such as weakness, arthralgia, depression, anxiety, and memory impairment [18]. Musculoskeletal dysfunction and muscle weakness can already be observed during hospitalization [19], with the viral infection directly contributing to dysfunction at cellular and tissue levels [20]. In addition, prolonged hospitalization and ICU stays with extended immobilization, inadequate food and protein intake and absorption, inflammation, low vitamin D levels, and acute illness can contribute to anabolic resistance and acute sarcopenia. This metabolic condition—characterized by reduced muscle protein synthesis—is further aggravated by aging, frailty, and obesity [21].

In the current COVID-19 scenario, social isolation requirements contributed to physical inactivity and sedentary behavior, accelerating muscle atrophy and reducing muscle function [22,23,24]. Accordingly, regular physical activity and appropriate exercise training are necessary to preserve muscle mass and prevent atrophy in older age [25]. Sarcopenia—defined as the progressive decline in muscle mass and function associated with aging and physical inactivity—if left untreated, is linked to poor quality of life and increased mortality [26,27]. In this context, inspiratory muscle training (IMT) has shown potential to promote mechanical and clinical improvements in patients affected by COVID-19 and has been considered a promising strategy for pulmonary rehabilitation in this population [28]. However, the available findings remain inconclusive, as they are largely based on very low- to low-quality evidence [29].

These challenges underscore the need for integrated and effective rehabilitation strategies for this population, with strength training representing a promising intervention to enhance physical and functional recovery after COVID-19. Therefore, this study aimed to investigate the impact of a rehabilitation program based on physical exercises in individuals who survived COVID-19 and developed severe disease

## 2. Materials and Methods

This study was a controlled, randomized clinical trial involving adults (≥18 years) of both sexes who survived COVID-19, were hospitalized, and developed severe disease in Campina Grande (PB). Participants were randomized using a blocked allocation procedure (two blocks) and assigned to one of two rehabilitation groups: multicomponent exercise (GEm) or multicomponent exercise combined with inspiratory muscle training (IMT; GEmTMI). Patients were recruited through public announcements on social media (Instagram Stories and feed). Given that the study was conducted during the period of strict public health restrictions related to the COVID-19 pandemic, which limited in-person attendance, reduced clinic capacity, and constrained recruitment and follow-up logistics, this trial was designed as a pragmatic, two-arm comparison of rehabilitation strategies rather than a no-intervention control trial; therefore, the absence of a control group should be interpreted as a key methodological limitation.

Eligible participants had two negative RT-PCR tests for COVID-19 performed at the hospitalization site, in accordance with discharge criteria for severe patients [30]. Severe disease was defined using the World Health Organization (WHO) clinical progression scale [31]. Participants had been hospitalized and managed in an intensive care unit (ICU), intermediate care unit, or ward and had received invasive mechanical ventilation (via orotracheal tube or tracheostomy) or non-invasive ventilatory support and oxygen therapy (high-flow nasal cannula or reservoir mask). All participants had been discharged from the hospital for at least 30 days before the start of rehabilitation. After receiving a detailed explanation of the study objectives and the exercise-based rehabilitation protocol, all participants provided written informed consent by signing the Informed Consent Form (ICF) approved by the ethics committee.

Participants were excluded if they had evidence of reinfection, rehospitalization, or hospital readmission [31]; were hemodynamically unstable (uncontrolled blood pressure and heart rate); had unstable medical conditions (e.g., cardiovascular or pulmonary disorders such as arrhythmias, decompensated atrial fibrillation, pulmonary thromboembolism, acute heart failure, pulmonary congestion, acute myocardial infarction, or cerebrovascular accident); had osteoarticular or neurological conditions limiting mobility (e.g., paraplegia, quadriplegia, amputation, or severe joint deformities); or had cognitive impairment or dementia that prevented completion of questionnaires and participation in the exercise protocol.

This study complied with Resolution No. 466/12 of the Brazilian National Health Council, which governs research involving human participants, and was submitted to the Research Ethics Committee of the UNIFACISA University Center. The protocol was approved under Opinion No. 4.619.192 and registered in the Brazilian Clinical Trials Registry (ReBEC) under UTN No. U1111-1264-9012 on 29 March 2021. Throughout the study, ethical standards were maintained, including appropriate care and monitoring of participants and protection of data confidentiality and privacy during data handling and after processing for publication. All individuals who voluntarily participated signed the Informed Consent Form (ICF) after reading and understanding its contents, including the study objectives, procedures, risks, and benefits. Participants were informed about all methodological stages (including group allocation) and the full assessment procedures. Each participant was advised of the right to withdraw at any time without any financial or other penalties. Participation was free of charge, and all study-related costs were covered by the researchers.

### 2.1. Sampling Plan

All participants underwent an initial assessment conducted by a physical education professional and a physiotherapist. At the outset of the study, government-imposed public health restrictions limited the number of individuals allowed in the rehabilitation clinic and therapy gym to reduce the risk of disease transmission. Therefore, recruitment and baseline characterization were conducted using a comprehensive remote assessment, combining medical record review with structured data collection and validated questionnaires. This included a review of medical records and length of hospitalization; collection of sociodemographic data (date of birth, sex, age, educational level, number of household members, and number of rooms in the home); and documentation of disease course (severity, duration of ICU or ward stay, use and duration of mechanical ventilation, surgical procedures, and medications used during treatment). Information on pre-existing comorbidities (cardiovascular, pulmonary, or metabolic diseases) and the use of related medications was also recorded. In addition, three previously validated questionnaires were administered to assess physical activity level (min/week), quality of life (SF-36), and post-COVID-19 functional status.

**Figure 1:**
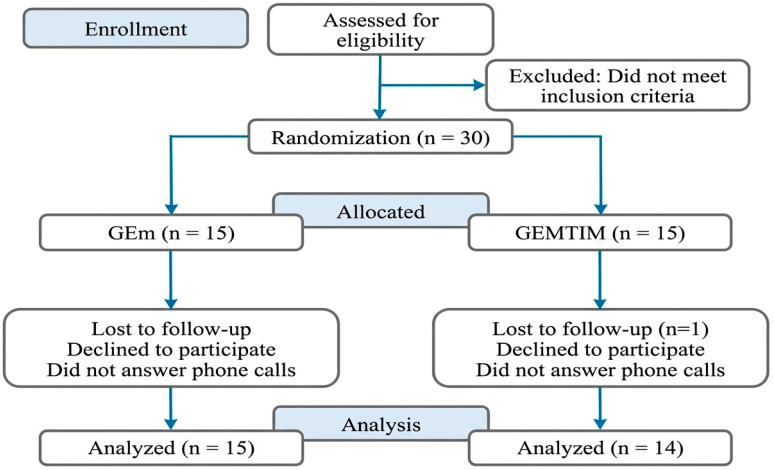
Flowchart of the sample and participant allocation into groups.

A formal a priori sample size calculation was not feasible because recruitment occurred during a period of strict public health restrictions, which limited clinic capacity and the number of eligible individuals who could be safely assessed in person; additionally, uncertainty regarding the expected effect sizes in this specific population of post-hospitalized patients with severe COVID-19 precluded the reliable specification of parameters for power estimation. Accordingly, the final sample reflects the number of eligible participants who could be recruited and followed within the operational constraints of the service during the study period. Moreover, guidance for external pilot randomized trials indicates that recommended sample sizes per treatment arm may range from approximately 10 to 75 participants, depending on the anticipated standardized effect size (e.g., ~10 per arm for large effects and larger samples for smaller effects), reinforcing that small samples are primarily appropriate for assessing feasibility and informing planning rather than for definitive inference of between-group differences [32]. Therefore, this small sample size should be interpreted as an important limitation, particularly with respect to detecting between-group differences. As shown in Figure 1 below.

### 2.2. Intervention

Rehabilitation sessions were conducted three times per week, with each session lasting 40–60 min, at the school clinic. All outcome measures were collected at baseline and after six weeks.

Both groups followed the same exercise-based rehabilitation protocol and differed only in the inclusion or absence of inspiratory muscle training (IMT). The exercise-only group performed multicomponent training (aerobic, resistance, and balance exercises) three times per week for 40–50 min. In the IMT group, a PowerBreathe device (HaB International Ltd., Hampshire, UK) was incorporated into the rehabilitation routine under physiotherapist supervision.

Aerobic training lasted 25–30 min and was performed on an ergometer selected by the participant (treadmill or cycle ergometer) to achieve the target heart-rate intensity ranges. Initial intensity was set at or below the heart rate corresponding to the first ventilatory threshold (VT1). Aerobic intensity was progressively increased according to the participant’s exercise tolerance until the second ventilatory threshold was reached. Perceived exertion was monitored using the modified Borg scale, ranging from 0 (no exertion) to 10 (maximal exertion), as proposed by [32].

Muscle-strengthening exercises lasted 10–15 min and included movements performed using body weight or external resistance (free weights, dumbbells, or elastic bands). Training targeted large muscle groups and consisted of 1–3 sets of 8–15 repetition maximum (RM), with 1–2 min rest intervals between sets [33,34]. Load progression was performed weekly with a 5–10% increase. If a participant was unable to complete the prescribed workload, the previous load was maintained, and a new attempt to increase the load was made the following week. Exercise intensity was additionally monitored using an analog perceived exertion scale for resistance training ranging from 0 (no exertion) to 10 (maximal effort), as proposed by [35].

Balance exercises lasted approximately 7–10 min and included motor tasks designed to challenge the center of gravity (e.g., tandem walking and walking on unstable surfaces), activities with reduced sensory input (e.g., sitting-to-standing and standing with eyes closed), and postural muscle challenges (e.g., toe walking and heel walking). These exercises were performed at either the beginning or the end of the session [36,37].

Inspiratory muscle training (IMT) to improve inspiratory muscle strength and endurance was performed by participants three times per week using a linear pressure-threshold loading device (Powerbreathe^®^ Classic line, HaB International Ltd., Hampshire, UK) and a nasal clip. Training began at 40–50% of maximal inspiratory pressure (PImax) and was progressively increased to 60–70% of PImax, up to the highest tolerable intensity. Participants performed 90 breaths per session, divided into three sets, with 1–2 min of rest between sets. PImax was reassessed every two weeks to adjust training intensity to the participant’s current capacity.

The training load was selected to provide the highest tolerable resistance while still allowing inspirations across the full vital capacity, thereby enhancing training specificity by applying a stimulus throughout the entire range of motion of the inspiratory muscles, including the lengths at which these muscles operate during exercise. Perceived respiratory effort (4–6 on the modified Borg 0–10 scale) was also used to guide decisions regarding load progression. IMT was performed either alone or in combination with resistance and balance exercises.

For safety, heart rate was monitored throughout each session using the Firstbeat Bodyguard 2 (Firstbeat Technologies Ltd., Jyväskylä, Finland). Blood pressure was measured by auscultation using an aneroid sphygmomanometer and stethoscope (Diasyst^®^, São Paulo, Brazil). Peripheral oxygen saturation was monitored using a pulse oximeter (TuffsatTM, São Paulo, Brazil). Capillary blood glucose was measured in participants with diabetes using a glucometer (Accu-Chek Nano, Roche, Germany).

If a participant exhibited peripheral oxygen saturation < 90% or developed symptoms such as palpitations, diaphoresis, chest tightness, or severe dyspnea, the session was immediately interrupted and the participant was placed at rest. The physiotherapist and the medical team then evaluated the participant to determine whether the session could be resumed, and supplemental oxygen was provided as needed.

### 2.3. Variables

#### 2.3.1. Physical Activity Level

Habitual physical activity level was assessed using the short form of the International Physical Activity Questionnaire (IPAQ) [38,39], which demonstrates moderate validity and reliability, with intraclass correlation coefficients (ICC) ranging from 0.33 to 0.62 [40]. Physical activity was estimated based on the duration and weekly frequency of walking and moderate- and vigorous-intensity activities performed across different domains. Total physical activity was expressed as minutes per week, and individuals accumulating ≥ 150 min/week were classified as physically active [41].

#### 2.3.2. Quality of Life

Quality of life was assessed using the Medical Outcomes Study 36-Item Short Form Health Survey (SF-36), which shows acceptable model fit, with comparative fit indices (CFI) ranging from 0.90 to 0.92 and a root mean square error of approximation (RMSEA) of 0.07. Validity is high, as indicated by significant correlations with other health-related quality-of-life measures, and reliability is also high, with Cronbach’s alpha coefficients often exceeding 0.90 [42]. The instrument has been previously validated in its Brazilian version [43].

The SF-36 comprises 36 items assessing eight domains: physical functioning (10 items), role limitations due to physical health (four items), bodily pain (two items), general health perceptions (five items), vitality (four items), social functioning (two items), role limitations due to emotional problems (three items), and mental health (five items), in addition to a comparative item on current health status relative to one year earlier. Domain scores range from 0 (worst) to 100 (best).

#### 2.3.3. Post-COVID-19 Functional Status Scale (PCFS)

Functional status was assessed using a recently published scale designed to capture relevant aspects of daily life during follow-up after infection [44]. This ordinal scale comprises six levels, ranging from 0 (“no symptoms”) to 5 (“death”), and spans the full spectrum of functional outcomes. It focuses on limitations in daily tasks and activities—both domestic and occupational/educational—as well as lifestyle changes. The scale assesses the domains of “constant care,” “activities of daily living (ADLs),” “instrumental activities of daily living (IADLs),” “participation in usual social roles,” and “symptom checklist.” It demonstrates significant correlations with other health-related quality-of-life measures and good reliability, with Cronbach’s alpha coefficients exceeding 0.76. Model fit indices were also acceptable, with comparative fit indices (CFI) ranging from 0.90 to 0.92 and a root mean square error of approximation (RMSEA) of 0.07 [45].

#### 2.3.4. Anthropometry and Body Composition

Body mass (kg) was measured using a Balmak^®^ Class III digital electronic (BALMAK SA, Athens, Greece) scale with a precision of 0.01 kg. Height was measured using a SANNY^®^ stadiometer (Personal Caprice Portable, São Paulo, Brazil) with a precision of 0.1 cm. Body mass index (BMI) was calculated as body mass divided by height squared (kg/m^2^). Body composition (fat and total body water percentage) was assessed by bioelectrical impedance analysis (BIA) using the Omron HBF-514C portable scale (OMRON, Kyoto, Japan), which applies a low-intensity, imperceptible electrical current.

#### 2.3.5. Dynamic Balance

Dynamic balance was assessed using the Timed Up and Go (TUG) test [46], which demonstrates high reliability and validity, with an intraclass correlation coefficient (ICC) of 0.82 and significant correlations with other mobility tests [47]. The TUG evaluates balance and functional mobility by measuring the time (in seconds) required for the participant to rise from a chair without using the arms, walk 3 m, turn, walk back, and sit down again. At the start of the test, the participant sat with the back against the backrest; at the end, the participant returned to the seated position with the back against the backrest. The participant was instructed to begin on the verbal cue “Go,” and timing started at the command and stopped when the participant sat back and reclined against the backrest [48].

#### 2.3.6. Functional Capacity

Functional capacity was assessed using the six-minute walk test (6MWT), with functional capacity quantified as the distance walked. The 6MWT has high validity for evaluating functional capacity in chronic diseases and shows significant correlations with other functional capacity measures. Test–retest reliability is high, with intraclass correlation coefficients (ICC) ranging from 0.80 to 0.97 [49]. The test was conducted in accordance with American Thoracic Society guidelines. Required equipment included a stopwatch, measuring tape, pulse oximeter (TuffsatTM, São Paulo, Brazil), heart rate monitor (735xt, Garmin^®^, Schaffhausen, Switzerland), sphygmomanometer (Diasyst^®^ Sphygmomanometer, São Paulo, Brazil), and stethoscope (Diasyst^®^, São Paulo, Brazil).

The 6MWT was performed in a 30 m corridor at the clinic. Vital signs—including blood pressure, heart rate, perceived exertion (0–10 analog scale) [32], and oxygen saturation—were measured before, during, and after the test. The outcome was the greatest distance covered in six minutes in a single trial.

Participants were instructed to stop the test if they experienced symptoms such as lower-limb pain, tachycardia, or respiratory discomfort. The pulse oximeter remained in place throughout the test to monitor peripheral oxygen saturation. The test was terminated if oxygen saturation decreased below 92% or if 90% of the age-predicted maximum heart rate was reached [36]. At the end of the test, the highest heart rate value was recorded as maximal heart rate (HRmax) and used to prescribe aerobic exercise intensity.

#### 2.3.7. Neuromuscular Fitness

Lower-limb muscle endurance was assessed using the 60 s sit-to-stand test from the Senior Fitness Test—Physical Fitness Test for Lower Limbs [50]. This test shows good reliability, with intraclass correlation coefficients (ICC) ranging from 0.76 to 0.93, as well as good concurrent validity, demonstrated by significant correlations with other physical fitness tests, and sensitivity to clinical change [51]. The test began with the participant seated on a chair with feet flat on the floor. Before the timed trial, the participant performed three repetitions to familiarize themselves with the task and then completed as many sit-to-stand repetitions as possible in 60 s.

Handgrip strength was assessed using a handgrip dynamometer test, which demonstrates high reliability (ICC: 0.90–0.97) and high validity, with strong correlations with other measures of muscle strength (e.g., isokinetic dynamometry). It is also sensitive to changes in functional status, supporting its use for monitoring therapeutic interventions [52]. Measurements were obtained using a digital dynamometer (EH101 model, Clear; adjustable and calibrated; range: 0–100 kgf). Participants were assessed in the standing position with the elbow fully extended and the arm slightly abducted, following the manufacturer’s instructions. Before testing, participants received standardized instructions and performed several practice contractions until comfortable with the procedure.

#### 2.3.8. Respiratory Muscle Strength

Maximal respiratory pressures were measured using an analog manovacuometer connected to a pressure line, a mouthpiece with a filter, and a connector with a 2 mm leak hole. Participants were seated with feet flat on the floor and an upright posture, without upper-limb support, and wore a nasal clip. They were instructed to perform the maximal inspiratory pressure (PImax) maneuver from residual volume (RV) to total lung capacity (TLC), followed by a maximal, sustained inspiratory effort. Maximal expiratory pressure (PEmax) was measured from TLC with a maximal, sustained expiratory effort.

At least three maneuvers were performed, with 1 min rest intervals between attempts, and reproducibility was defined as a ≤10% variation between maneuvers. The highest value obtained from the acceptable maneuvers was recorded. Predicted values for PImax and PEmax were calculated for each participant using equations validated for the Brazilian population.

#### 2.3.9. Cardiopulmonary Exercise Teste (CPET)

Before the cardiopulmonary exercise test (CPET) to determine peak VO_2_, participants were informed about the procedures and instructed to exercise to volitional exhaustion. A standard 12-lead ECG and peripheral oxygen saturation were recorded at rest and at the end of each workload stage using a Vyntus^®^ ECG system (Vyaire, Leipzig, Germany). The test could be terminated if any criterion for test termination, according to current guidelines, was met [53,54,55]. Blood pressure was measured by auscultation at rest, at the end of each CPET stage, and during active recovery using a sphygmomanometer (Diasyst^®^, São Paulo, Brazil) and stethoscope (Diasyst^®^, São Paulo, Brazil). Oxygen saturation was continuously monitored throughout the test using a pulse oximeter (TuffsatTM, São Paulo, Brazil). The CPET was conducted by a sports physician (member of the multidisciplinary team), with support from a physical education professional and a physiotherapist.

To familiarize participants with treadmill walking, the test began on a flat, motorized treadmill (Movement, R7 127V), during which participants practiced walking without holding the handrails. For participants who could walk safely, a 10 min warm-up was performed at a fixed speed of 3–6 km/h and 0% grade. After warm-up, a face mask was fitted for metabolic measurements using the Vyntus^®^ CPX system (Vyaire, Leipzig, Germany).

The A ramp protocol was used, starting at 4.0 km/h and 1.0% grade, with speed increased by 0.5 km/h each minute until peak VO_2_ was achieved. The protocol was designed to elicit peak VO_2_ within 8–12 min, in line with recommendations [56]. The test was terminated upon volitional exhaustion or if any of the following occurred: diastolic blood pressure (DBP) increased to 120 mmHg; systolic blood pressure (SBP) decreased persistently by >10 mmHg; SBP increased disproportionately to 260 mmHg with rising workload; chest discomfort with increasing workload or accompanied by ECG changes suggestive of ischemia; ataxia, dizziness, pallor, cyanosis, or presyncope; dyspnea disproportionate to exercise intensity; ST-segment depression of 0.3 mV (2.5 mm) or ST-segment elevation of 0.2 mV (2.0 mm) in leads without a Q wave; complex ventricular arrhythmias; onset of non-sustained or sustained supraventricular tachycardia; atrial tachycardia; atrial fibrillation; second- or third-degree atrioventricular block; or signs of left ventricular failure [57]. Because of the high cost and logistical constraints of CPET, it was performed only at baseline to determine training intensities prior to the rehabilitation program.

### 2.4. Statistical Analysis

Results are presented as mean ± standard deviation [95% CI] or as proportions. Data normality was assessed using the Shapiro–Wilk test due to the small sample size. Within-group pre- to post-rehabilitation changes were analyzed using the two-tailed Wilcoxon signed-rank test for nonparametric continuous data and the chi-square test for categorical data, as appropriate, whereas paired *t*-tests were used for parametric data. Between-group comparisons were performed using the Mann–Whitney U test for nonparametric data and independent-samples *t*-tests for parametric data. Statistical significance was set at *p* < 0.05. All analyses were conducted in SPSS version 20 (IBM, Armonk, NY, USA).

## 3. Results

The 30 eligible patients were allocated to two rehabilitation groups: the physical exercise group (GEm; *n* = 15) and the physical exercise plus inspiratory muscle training group (GEmTMI; *n* = 14). One patient was excluded from the study due to absences related to transportation difficulties and was therefore not included in the final evaluation. Participants’ demographic characteristics are presented in Table 1.

No adverse events were reported during the program. SpO_2_ remained above 90% in all participants throughout the training sessions. Data from 29 participants—assigned to the GEm (*n* = 15) and GEmTMI (*n* = 14) groups—were analyzed to characterize baseline demographic and anthropometric features that could influence study outcomes. As shown in Table 1, there was no statistically significant evidence of between-group differences in age, sex, weight, height, BMI, or disease prevalence. Although not reported in Table 1, educational level was also assessed; the chi-square test (χ^2^ = 4.95) indicated no significant difference in its distribution between groups (*p* = 0.66).

As shown in Table 2, both groups improved across several domains, particularly social functioning and physical limitations; however, many of these improvements did not reach statistical significance.

The results presented in Table 3 indicate that the intensive mobility intervention (TMI) yielded significant benefits, including symptom reduction; improvements in instrumental activities of daily living and social role functioning; and increases in physical activity and energy expenditure among participants.

The results presented in Table 3 indicate that the intensive mobility intervention (IMT) yielded significant benefits, including symptom reduction; improvements in instrumental activities of daily living and social role functioning; and increases in physical activity and energy expenditure among participants. Although both groups improved, the magnitude of change was greater in the GEmTMI group. These findings suggest that IMT may be an effective intervention for improving quality of life and functional status in similar populations. The participants’ cardiopulmonary exercise capacity, including heart rate at the first and second ventilatory thresholds (VT1 and VT2), peak heart rate, and peak oxygen uptake (VO_2_peak; mL·kg^−1^·min^−1^), is presented in Table 4.

The parameters evaluated and presented in Table 5 were TC6 (six-minute walk test), TSL (time to stand and sit), TSL 1min (time to stand and sit in 1 min), TUG (timed up and go test), Right and Left Handgrip, PEMÁX (maximum expiratory pressure), and PImax (maximum inspiratory pressure).

The parameters evaluated and reported in Table 5 were the six-minute walk test (6MWT), time to stand and sit (TSL), one-minute stand-and-sit test (TSL 1 min), the timed up-and-go test (TUG), right and left handgrip strength, maximal expiratory pressure (PEmax), and maximal inspiratory pressure (PImax). Measurements were obtained before and after the interventions and were compared both within groups and between groups at the two time points. Both the GEm and GEmTMI groups showed significant post-intervention improvements across all assessed parameters. No significant between-group differences were observed, suggesting comparable effectiveness of the interventions. Overall, the findings underscore the effectiveness of both training programs in improving functional capacity, muscle strength, and endurance in the participants.

## 4. Discussion

At the start of rehabilitation (following the acute phase), the GEm and GEmTMI groups were comparable. The mean age (50.67 ± 11.96 years) was consistent with that reported across 11 post-COVID-19 rehabilitation studies (55.81 ± 8.82 years) [58,59,60,61,62,63,64,65,66,67,68]. Other baseline characteristics (Table 1)—including sex, weight, height, and the prevalence of non-communicable diseases (NCDs)—were also well balanced between groups, with obesity being the most prevalent condition (65.52%). Obesity is associated with hypertension, type 2 diabetes, cardiovascular disease, stroke, and cancer [69], which increases clinical vulnerability to SARS-CoV-2 infection and worsens prognosis [70]. Obese patients have a 2.1-fold higher risk of hospitalization, a 2.6-fold higher risk of ICU admission, and a 3.7-fold higher risk of death [71], and overweight/obesity is highly prevalent among hospitalized individuals [70]. The pro-inflammatory and immunomodulatory effects of adipose tissue—particularly visceral fat—may further exacerbate disease progression [72,73]. Accordingly, rehabilitation strategies should incorporate weight-management components to reduce the risks of respiratory failure and systemic inflammation.

During the peak VO_2_ test, 29 patients achieved maximal effort without complications; however, exercise capacity was markedly reduced (16.14 ± 5.52 mL·kg^−1^·min^−1^), below the 20 mL·kg^−1^·min^−1^ threshold often cited as necessary for independent activities of daily living [74]. This impairment may reflect COVID-19–related stress and mitochondrial dysfunction, including reduced oxidative phosphorylation and mitochondrial density [75,76,77,78,79,80,81], as well as possible activation of catabolic pathways linked to skeletal muscle atrophy [33,82,83]. After six weeks, meaningful functional gains were observed. Mean 6MWT distance increased from 378.44 ± 103.06 m to 501.33 ± 76.95 m (+122.89 m; +32.47%), consistent with previous reports [84]. This improvement likely reflects reductions in fatigue and dyspnea and, importantly, supports greater autonomy and self-efficacy, thereby benefiting social functioning and quality of life [85]. Performance on the sit-to-stand test (STST) increased by 37.89%, from 19.89 ± 6.23 to 27.30 ± 7.40 repetitions (+7.41), corroborating the improvements observed in the 6MWT. The more pronounced perception of dyspnea relative to lower-limb fatigue is consistent with the predominant pulmonary contribution to functional limitation after COVID-19 [86], supporting the integration of aerobic training, resistance exercise, and respiratory conditioning within rehabilitation protocols.

A meaningful improvement was observed in the Timed Up and Go (TUG) test, with times decreasing from 9.59 ± 3.68 to 7.00 ± 1.88 s. Notably, all six participants who initially exceeded 12 s—a threshold indicating high fall risk according to CDC/STEADI—fell below this cutoff after the intervention [87]. In long COVID and post-critical illness, prolonged TUG and dual-task TUG (DT-TUG) times are common [88,89]. On average, post-COVID patients take longer to complete sit-to-stand (STS), walking, and TUG tests than healthy individuals [90], likely due to central and peripheral neurological changes that impair gait symmetry [91,92], as well as muscle weakness, exercise intolerance, and histological atrophy [93]. These findings support the clinical relevance of rehabilitation for mobility and safety while remaining consistent with the functional tests reported.

Handgrip strength also increased, from 28.81 ± 10.31 to 34.70 ± 11.37 kg in the right hand and from 26.67 ± 9.62 to 32.63 ± 10.61 kg in the left hand—clinically relevant markers of health and functional status [94] that are often reduced after COVID-19 hospitalization [95,96,97]. Aerobic and resistance exercise can reduce dyspnea and restore strength [98], and early rehabilitation may help prevent critical illness neuromyopathy (CINM) and ICU-acquired weakness (ICU-AW) [99,100,101]. Importantly, muscle quality—captured by markers such as myosteatosis and intermuscular adipose tissue (IMAT)—may be more strongly associated with physical performance than muscle mass alone [102,103]. Elevated IMAT, often linked to chronic inflammation and altered lipid metabolism, is a marker of poor muscle quality and may pre-exist in hospitalized patients, thereby impairing functional recovery [104,105]. Accordingly, the improvements observed here may reflect not only gains in strength but also potential rehabilitation-related improvements in neuromuscular performance and muscle quality.

With respect to respiratory function, respiratory muscle strength is critical for sustaining functional gains. Rehabilitation increased maximal inspiratory pressure (PImax) and maximal expiratory pressure (PEmax), which is clinically relevant for reducing dyspnea and supporting mobility, although some studies note limited statistical power due to small sample sizes [106]. Overall, the pattern of improvement underscores the interdependence of respiratory conditioning and physical performance: greater respiratory strength may reduce perceived exertion during daily activities, which is consistent with improvements in psychosocial outcomes, including social functioning on the SF-36.

The combination of inspiratory muscle training (IMT) and aerobic exercise improves respiratory muscle strength, with significant increases in maximal inspiratory pressure (PImax) reported in the literature [107], as well as cardiovascular benefits in hypertensive individuals [108]. Tele-supervised home-based protocols can expand access and have demonstrated effectiveness [61]. Pulmonary rehabilitation reduces dyspnea, improves exercise capacity, and alleviates fatigue; meta-analyses indicate greater improvements in pulmonary function and quality of life compared with no intervention [109]. Physical training may also accelerate functional recovery and improve spirometric parameters such as forced vital capacity (FVC) and forced expiratory volume in 1 s (FEV_1_) [110], a hypothesis further supported by the potential anti-inflammatory effects of exercise [110]. In this context, evidence from an Individual Rehabilitation Project (IRP) grounded in early re-educational interventions in hospitalized COVID-19 patients also reported improvements in dyspnea and autonomy in activities of daily living and muscle strength, while highlighting that a no-rehabilitation control group was not feasible for clinical and ethical reasons. This evidence reinforces the importance of early intervention in the post-COVID-19 recovery process [111].

In our study, PImax and PEmax increased after rehabilitation in both the GEm and GEmTMI groups. The absence of statistically significant between-group differences may be explained by the non-specific recruitment of respiratory muscles during whole-body exercise, which could attenuate any incremental effect of IMT. In addition, the small sample size limits the precision of between-group comparisons and reduces power to detect modest incremental effects—an aspect that should be interpreted as an important constraint rather than evidence of equivalence. Nevertheless, these findings are consistent with evidence that both resistance and aerobic training can improve PImax and PEmax [112,113,114].

Taken together, improvements in peak VO_2_, 6MWT distance, TUG performance, and STST repetitions likely contributed to reduced fatigue and dyspnea while enhancing autonomy. These physical gains were accompanied by higher SF-36 social functioning scores and a lower symptom burden (PCFS). Concurrently, restoration of respiratory muscle strength (PImax and PEmax) may have supported functional performance and reduced perceived exertion. Overall, structured interventions integrating aerobic, resistance, and respiratory training appear to contribute to multidimensional recovery, while conclusions about the incremental contribution of IMT should remain cautious given the study design and sample size.

The findings of this study underscore that post-COVID-19 rehabilitation should be viewed as a multidimensional process in which functional, psychosocial, and respiratory gains are interrelated and mutually reinforcing. Improvements in exercise capacity (peak VO_2_, 6MWT, and STST), functional mobility (TUG), and peripheral muscle strength (handgrip strength) are consistent with structured interventions that can support autonomy and reduce persistent symptoms such as fatigue and dyspnea. These physical gains may also translate into psychosocial benefits, reflected in higher SF-36 social functioning scores and fewer functional limitations on the PCFS, suggesting greater confidence, social participation, and quality of life.

Concurrently, respiratory improvements—evidenced by increases in PImax and PEmax—reinforce the central role of pulmonary function in sustaining physical capacity and reducing perceived exertion, thereby linking physiological recovery to psychosocial reintegration. Together, these results support the clinical relevance of rehabilitation programs that integrate aerobic, resistance, and respiratory training in minimizing sequelae, promoting comprehensive recovery, and potentially mitigating the long-term impact of infection.

Although the study has methodological limitations, including a small sample size and the absence of a control group, data collection occurred during a period of strict restrictions on gatherings, which constrained recruitment and service capacity. Therefore, the results should be interpreted cautiously because improvements may partially reflect natural recovery over time in addition to intervention-related effects. Moreover, while the lack of a no-intervention control arm remains a key limitation for causal inference, this design choice is consistent with the clinical and ethical rationale reported in hospital-based COVID-19 rehabilitation research, where patients are not left without rehabilitation assistance.

Nevertheless, the findings highlight the importance of establishing post-COVID-19 rehabilitation as a priority within longitudinal care pathways. Beyond improving patients’ physical and mental health, such interventions provide a basis for future research to clarify recovery mechanisms, quantify the incremental contribution of IMT, and expand therapeutic options.

## 5. Conclusions

This study demonstrated that structured physical exercise programs can support recovery in COVID-19 survivors, particularly those who experienced severe disease. Both groups, with or without inspiratory muscle training (IMT), showed significant improvements in physical capacity, strength, and endurance. Although between-group differences were not statistically significant, the findings suggest clinically meaningful benefits, especially in social functioning and reductions in physical limitations. Nevertheless, these results should be interpreted with caution, given the possibility that some improvements may reflect the natural course of recovery.

## Figures and Tables

**Table 1:** Demographic characteristics of the patients Total (GEm + GEmTMI), GEm, and GEmTMI for men (M) and women (F).

Variables	Total *n* = 29	GEm *n* = 15	GEmTMI *n* = 14	*p*-Value (Gem vs. GEmTMI)
Age (years)	50.67 ± 11.96 [47.50;53.83]	51.80 ± 13.54	49.36 ± 10.86	0.588
Gender (M/F)	13/16	6/9	7/7	0.468
Weight (Kg)	93.35 ± 20.99 [84.87;101.82]	92.04 ± 24.26	83.33 ± 21.73	0.905
Height (cm)	166.07 ± 13.64 [161.56;170.58]	159.92 ± 12.64	163.08 ± 9.38	0.128
BMI (Kg/m^2^)	33.77 ± 6.96 [30.96;36.58]	36.01 ± 9.28	31.25 ± 7.14	0.655
People in the household	2.66 ± 1.52 [2.08;3.23]	2.73 ± 1.67	2.57 ± 1.40	0.78
Hypertension (*n*)	14	8	6	0.048 *
Obesity (*n*)	19	10	9	
Type 2 Diabetes (*n*)	7	4	3	
Asthma (*n*)	6	3	3	
Arthritis (*n*)	5	3	2	

Legend: Baseline sociodemographic and clinical characteristics of the participants (total *n* = 29) allocated to the GEm (*n* = 15) and GEmTMI (*n* = 14) groups. Data are presented as mean ± standard deviation and 95% confidence interval (95% CI) or as counts/proportions. *p*-values refer to between-group comparisons (GEm vs. GEmTMI). * Indicates a statistically significant difference (*p* < 0.05). BMI, body mass index; M, male; F, female.

**Table 2:** Quality of Life (SF 36) of the patients Total (GEm + GEmTMI), GEm, and GEmTMI.

Variables	Total *n* = 29			GEm *n* = 15			GEMTMI *n* = 14			GEm vs. GEmTMI/1ª	GEm vs. GEmTMI/2ª
SF-36	1ª	2ª	*p*	1ª	2ª	*p*	1ª	2ª	*p*	*p*	*p*
Physical Functioning	43.33 × 30.16 [31.40/55.26]	48.28 × 25.87 [38.02/58.50]	0.353	48.93 × 28.57 [32.43/65.42]	48.43 × 23.66 [34.77/62.09	0.875	37.31 × 31.80 [18.09/55.52]	48.08 × 29.05 [30.52/55.63]	0.375	0.253	0.973
Role Limitations due to Physical Health	16.67 × 23.59 [5.36/27.99]	45.78 × 55.88 [31.59/59.97]	0.006 *	12.50 × 27.30 [2.26/28.26]	38.29 × 34.94 [18.17/58.40]	0.103	21.15 × 30.36 [2.81/39.50]	53.85 × 36.58 [31.74/75.89]	0.023 *	0.31	0.293
Pain	45.00 × 35.48 [30.99/59.04]	61.70 × 23.97 [32.22/71.18]	0.061	38.50 × 31.61 [20.25/56.76]	60.71 × 25.01 [46.28/75.15]	0.091	52.00 × 90.28 [28.27/75.73]	62.77 × 23.76 [48.41/77.18]	0.408	0.333	0.829
General Health	50.56 × 15.61 [45.17/55.64]	49.81 × 14.17 [44.21/55.42]	0.837	46.79 × 7.90 [42.46/51.11]	48.57 × 11.84 [41.74/55.41]	0.658	54.62 × 17.80 [44.04/65.19]	51.15 × 6.73 [41.06/61.26]	0.583	0.155	0.645
Vitality	43.15 × 15.75 [36.91/49.38]	47.59 × 9.74 [43.74/51.48]	0.262	45.36 × 16.31 [55.65/55.05]	45.36 × 9.30 [39.99/50.72]	1.000	40.77 × 14.84 [31.80/40.74]	50.00 × 10.00 [43.96/66.08]	0.128	0.461	0.176
Social Functioning	51.81 × 32.04 [39.14/64.49]	74.11 × 24.95 [64.24/83.98]	0.009 *	50.99 × 33.74 [31.45/70.41]	70.57 × 25.71 [55.73/85.42]	0.073	52.77 × 31.45 [33.76/71.76]	77.92 × 24.33 [63.10/92.78]	0.076	0.885	0.424
Role Limitations due to Emotional Problems	46.96 × 45.57 [28.94/94.59]	56.81 × 45.14 [98.96/74.67]	0.442	54.86 × 44.57 [29.12/50.50]	42.93 × 42.23 [18.51/97.34]	0.359	38.46 × 46.86 [10.15/66.78]	71.77 × 44.84 [44.07/98.87]	0.086	0.42	0.055
Mental Health	58.52 × 18.10 [51.36/56.88]	62.67 × 18.09 [56.70/88.64]	0.330	59.43 × 19.33 [28.27/70.59]	60.00 × 10.29 [54.06/66.94]	0.921	57.54 × 17.40 [47.02/88.05]	65.54 × 19.01 [54.05/77.03]	0.223	0.792	0.351

Legend: SF-36 domain scores at the 1st and 2nd assessments for the total sample (*n* = 29) and for the GEm (*n* = 15) and GEMTMI (*n* = 14) groups. Data are presented as mean ± standard deviation [95% confidence interval] or proportions. Within-group *p*-values compare the 1st vs. 2nd assessments, and between-group *p*-values compare GEm vs. GEmTMI at each assessment. * indicates a statistically significant difference (*p* < 0.05).

**Table 3:** Post-COVID-19 Functional State Scale (PCFS) and physical activity level (IPAQ) of the patients Total (GEm + GEmTMI), GEm, and GEmTMI.

Variables	Total *n* = 29			GEm *n* = 15			GEmTMI *n* = 14			GEm vs. GEmTMI/1º	GEm vs. GEmTMI/2º
	1º	2º	*p*	1º	2º	*p*	1º	2º	*p*	*p*	*p*
PCSF_symp	1.76 ± 1.21 [1.29/2.23]	1.09 ± 0.97 [0.71/1.46]	0.023 *	1.55 ± 1.07 [0.93/2.17]	1.28 ± 0.99 [0.71/1.85]	0.544	1.98 ± 1.34 [1.21/2.75]	0.89 ± 0.94 [0.35/1.44]	0.013 *	0.417	0.275
PCSF_IADL	1.45 ± 1.81 [0.74/2.15]	0.59 ± 1.17 [0.14/1.04]	0.040 *	1.46 ± 1.79 [0.43/2.50]	0.46 ± 0.93 [0.7/1.00]	0.122	1.43 ± 1.90 [0.33/2.53]	0.71 ± 1.40 [0.09/1.52]	0.307	0.903	0.678
PCSF_Soc	1.55 ± 1.24 [1.06/2.03]	0.92 ± 1.05 [0.52/1.33]	0.030 *	1.18 ± 1.18 [0.51/1.86]	1.06 ± 1.07 [0.44/1.68]	0.624	1.91 ± 1.24 [1.20/2.63]	0.79 ± 1.04 [0.18/1.39]	0.014 *	0.128	0.431
PCSF_Total	1.18 ± 0.92 [0.82/1.54]	0.64 ± 0.79 [0.33/0.94]	0.011 *	1.09 ± 0.97 [0.52/1.65]	0.63 ± 0.69 [0.23/1.03]	0.158	1.39 ± 0.90 [0.76/1.80]	0.64 ± 0.91 [0.12/1.17]	0.026 *	0.597	0.835
Weekly Physical Activity Minutes (IPAQ)	54.14 ± 51.85 [34.04/74.25]	102.82 ± 75.31 [73.62/132.02]	0.012 *	67.57 ± 59.75 [33.08/102.07]	69.21 ± 62.91 [32.89/105.54]	0.972	40.71 ± 40.33 [17.43/64.00]	136.43 ± 73.42 [94.04/178.82]	0.002 *	0.308	0.002 *
Weekly MET minutes (IPAQ)	730.75 ± 765.64 [433.87/1027.63]	1652.95 ± 1725.65 [983.81/2322.08]	0.012 *	1034.29 ± 886.47 [522.45/1546.12]	1256.18 ± 2257.08 [47.018/2559.38]	0.650	427.21 ± 482.92 [148.38/706.05]	2049.71 ± 866.79 [1549.25/2550.18]	0.001 *	0.106	0.002 *

Legend: PCFS outcomes (symptoms, instrumental activities of daily living, social domain, and total score) and weekly physical activity assessed by the IPAQ (weekly physical activity minutes and weekly MET-minutes) for the total sample (*n* = 29) and for the GEm (*n* = 15) and GEmTMI (*n* = 14) groups, evaluated at the 1st and 2nd assessments. Data are presented as mean ± standard deviation and 95% confidence interval (95% CI). * indicates a statistically significant difference (*p* < 0.05).

**Table 4:** Cardiopulmonary exercise capacity of the patients Total (GEm + GEmTMI), GEm, and GEmTMI.

Variables	Total *n* = 29	GEm *n* = 15	GEmTMI *n* = 14	GEm vs. GEmTMI 1º
FC_LV1	119.14 ± 13.71 [111.23/127.06]	122.00 ± 16.69 [101.28/142.72]	118.00 ± 13.91 [100.73/135.27]	0.484
FC_LV2	131.50 ± 19.13 [120.46/142.55]	136.20 ± 25.59 [104.43/167.97]	129.60 ± 16.70 [108.87/150.33]	0.451
FC_Peak	140.36 ± 24.24 [126.36/154.35]	145.60 ± 30.33 [107.94/183.26]	141.40 ± 24.64 [110.80/171.99]	0.896
mL/Kg/min	16.14 ± 5.52 [12.96/19.33]	20.70 ± 6.78 [12.28/29.12]	13.22 ± 2.61 [9.98/16.46]	0.450

Legend: Heart rate (HR) at ventilatory threshold 1 (VT1), ventilatory threshold 2 (VT2), and peak exercise, as well as peak oxygen uptake (VO_2_peak; mL·kg^−1^·min^−1^), for the total sample (*n* = 29) and for the GEm (*n* = 15) and GEmTMI (*n* = 14) groups. Data are presented as mean ± standard deviation and 95% confidence interval (95% CI). *p*-values refer to between-group comparisons (GEm vs. GEmTMI).

**Table 5:** Functional tests of the patients Total (GEm + GEmTMI), GEm, and GEmTMI.

Variables	Total *n* = 29			GEm *n* = 15			GEmTMI *n* = 14			GEm vs. GEmTMI 1º	GEm vs. GEmTMI 2º
	1º	2º	*p*	1º	2º	*p*	1º	2º	*p*	*p*	*p*
TC6 (m)	378.44 ± 103.06 [337.67/419.21]	501.33 ± 76.95 [470.89/531.77]	0.000 *	344.0 ± 99.67 [286.45/401.55]	480.50 ± 81.84 [433.25/527.75]	0.006 *	415.54 ± 96.84 [357.02/474.06]	523.77 ± 67.24 [483.14/564.40]	0.005 *	0.043 *	0.094
TSL (5Rep-seg)	15.56 ± 5.21 [13.50/17.61]	11.30 ± 2.71 [10.22/12.37]	0.000 *	16.07 ± 5.59 [12.84/19.30]	11.43 ± 2.85 [9.78/13.07]	0.007 *	15.00 ± 4.92 [12.03/17.97]	11.15 ± 2.67 [9.54/12.77]	0.006 *	0.503	0.608
TSL 1 min	19.89 ± 6.23 [17.42/22.35]	27.30 ± 7.40 [24.37/30.23]	0.000 *	18.29 ± 5.30 [15.23/21.35]	27.21 ± 8.41 [22.36/32.07]	0.001 *	21.62 ± 6.89 [17.45/25.78]	27.38 ± 6.49 [23.46/31.31]	0.000 *	0.107	0.677
TUG	9.59 ± 3.68 [8.14/11.05]	7.00 ± 1.88 [6.26/7.74]	0.000 *	10.50 ± 4.62 [7.83/13.17]	7.57 ± 2.34 [6.22/8.92]	0.002 *	8.62 ± 2.06 [7.37/9.86]	6.38 ± 0.96 [5.80/6.97]	0.000 *	0.416	0.089
Right Hand Grip	28.81 ± 10.31 [24.74/32.89]	34.70 ± 11.37 [30.21/39.20]	0.000 *	27.64 ± 6.69 [23.78/31.50]	33.36 ± 8.51 [28.44/38.27]	0.000 *	30.08 ± 13.36 [22.01/38.15]	36.15 ± 14.04 [27.67/44.64]	0.000 *	0.477	0.444
Left Hand Grip	26.67 ± 9.62 [22.86/30.47]	32.63 ± 10.61 [28.43/36.83]	0.000 *	24.71 ± 7.40 [20.44/28.99]	30.57 ± 9.16 [25.28/35.86]	0.000 *	28.77 ± 11.49 [21.83/35.71]	34.85 ± 11.95 [27.62/42.07]	0.000 *	0.239	0.235
PeMax	101.52 ± 33.90 [88.11/114.93]	120.00 ± 30.41 [107.97/132.03]	0.000 *	97.86 ± 36.20 [76.96/118.76]	116.79 ± 33.89 [97.22/136.35]	0.002 *	105.46 ± 32.21 [85.99/124.93]	123.46 ± 27.11 [107.08/139.84]	0.015 *	0.421	0.378
PiMax	107.59 ± 56.49 [85.25/129.94]	147.22 ± 56.49 [124.88/169.57]	0.000 *	116.43 ± 64.88 [78.97/153.89]	153.21 ± 69.93 [112.84/193.59]	0.001 *	98.08 ± 46.53 [69.96/126.19]	140.77 ± 39.10 [117.14/164.39]	0.003 *	0.316	0.511

Legend: Functional test outcomes for the total sample (*n* = 29) and for the GEm (*n* = 15) and GEmTMI (*n* = 14) groups at the 1st and 2nd assessments. The table includes the 6-min walk test (6MWT), sit-to-stand test (STS; repetitions/30 s and 1 min), timed up and go (TUG), right and left handgrip strength, and maximal inspiratory and expiratory pressures (PImax and PEmax). Data are presented as mean ± standard deviation and 95% confidence interval (95% CI). *p*-values refer to within-group comparisons (1st vs. 2nd) and between-group comparisons (GEm vs. GEmTMI) at each assessment. * indicates a statistically significant difference (*p* < 0.05).

## Data Availability

The original contributions presented in this study are included in the article. For further information, please contact the corresponding authors.
